# COVID-19, an Opportunity for Developing Countries?

**DOI:** 10.3389/fpubh.2020.548708

**Published:** 2020-11-23

**Authors:** Lee Smith, Nicola Veronese, Vincenzo Racalbuto, Damiano Pizzol

**Affiliations:** ^1^The Cambridge Centre for Sport and Exercise Sciences, Anglia Ruskin University, Cambridge, United Kingdom; ^2^Geriatric Unit, Department of Internal Medicine and Geriatrics, University of Palermo, Palermo, Italy; ^3^Italian Agency for Development Cooperation - Khartoum, Khartoum, Sudan

**Keywords:** COVID-19, coronavirus infections, low-and lower-middle-income countries, coronavirus, perspective

## Abstract

The COVID-19 outbreak was declared by the World Health Organization (WHO) as global pandemic in March 2020. Considering the necessity to implement rapid response to control the pandemic and the fragility and the state of need of low income countries, it will be mandatory to develop a global approach in order to reduce the spread of infection and the creation of community viral reservoirs. So far, we could hypothesize a worst case scenario in which when the COVID-19 outbreak hits a peak in Africa and in low-income countries, the majority of such countries will be unprepared, with low resources allocated for affording the viral emergency and the consequences will be catastrophic with no lesson learnt. In the best case scenario, the COVID-19 will not affect Africa or South America on a large scale and, if the prevention measures will be implemented, we could register a lower incidence of hygiene linked diseases that still represent leading causes of death.

## Correspondence

The COVID-19 outbreak was declared, by the World Health Organization (WHO), a global pandemic in March 2020. The number of confirmed cases is increasing worldwide and after Asian and European regions, a steep increase is now being observed in low-income countries (1). It is being recommended that to reduce the spread of the virus SARS-Cov-2 that leads to the disease COVID-19, a rapid implementation of public health response strategies is required including isolation, quarantine, social distancing, and community containment measures (2). Considering the necessity of implementing a rapid response to control the pandemic, as well as the fragility and state of need of low income countries, it will be mandatory to develop a general approach to reduce the infection spreading and the creation of community viral reservoirs. Importantly, the African Task Force for Coronavirus Preparedness and Response (AFTCOR) has now been established. If successful, it may provide a model for low-and-middle income countries to follow. This task force includes six main lines: risk communication; prevention and control activities in health care centers and hospitals; surveillance, including screening at the borders and customs; labs testing and sub-typing; treatment of positive patients; and supply chain management (3). Prevention measures recommended by WHO include: (I) Regular and thorough hand-washing with an alcohol-based solution; (II) Avoid touching eyes, nose and mouth; (III) Accurate respiratory hygiene: in case of a cough or sneeze it is important to cover mouth and nose with bent elbow or tissue; (IV) in case of fever, cough and difficulty breathing, it is important to consult a physician early; (V) correct information, paying attention to fake news and following health care advice; (VI) maintenance of security distance (one meter or three feet) from people coughing or sneezing (4). The majority of these are basic good standards for hygiene and should routinely be applied worldwide. However, such standards are not common especially in low-income countries. Thus, if the COVID-19 outbreak results in the routine introduction of these measures, cross-over benefit may occur; that is, we could observe reductions in others diseases linked to poor sanitation conditions such as pneumonia, gastroenteritis, diarrhea, dysentery, hepatitis A, cholera, typhoid, polio, and skin infections.

Therefore, according to the knowledge we have so far, we could conjecture a worst- and a best-case scenario. In the worst case scenario, when the COVID-19 outbreak hits a peak in Africa and in low-income countries, the majority of such countries will be unprepared, with low resources allocated for affording the viral emergency and the consequences will be catastrophic with no lesson learnt. This would be the defeat of global health in an International Community that would show its slowness and cumbersome nature. At the beginning, in the best case scenario, it was hypothesized that COVID-19 would have not impacted so deeply sub-Saharan Africa and South America, similarly to the global outbreak of SARS-CoV in February 2003, suggesting that the spread of these viruses is more likely in the cold season and, thus, the southern hemisphere could be affected later or not at all (3). Unfortunately, South America is currently heavily affected showing substantial difference from 2003. In sub-Saharan Africa, on the contrary, although precise data are missing, it seems to have less impact, this may be due to other climate-related differences including outdoor life, the effect of hot and solar light in reducing the survival of COVID-19 on external surfaces and, different innate immunity of different ethnicities (3). In addition to this hopeful low impact, if the prevention measures are implemented, we could register a lower incidence of hygiene-linked diseases that still represent leading causes of death (5).

In conclusion, despite the difficulties and the negative effects that this virus will have worldwide, we are confident that, especially in sub-Saharan Africa, it could have some positive collateral effects owing to the relating public health messages.

## Data Availability Statement

The original contributions presented in the study are included in the article/supplementary materials, further inquiries can be directed to the corresponding author/s.

## Author Contributions

LS and DP prepared the first draft. NV and VR made the final revision. All authors contributed to the article and approved the submitted version.

## Conflict of Interest

The authors declare that the research was conducted in the absence of any commercial or financial relationships that could be construed as a potential conflict of interest.
